# Is remnant preservation in anterior cruciate ligament reconstruction superior to the standard technique? An overview of systematic reviews

**DOI:** 10.1186/s12891-023-07030-4

**Published:** 2023-11-24

**Authors:** Yunsong Zhang, Xiangyu Xiao, Wei Deng, Jianyu Wang, Hongwei Gao, Jicheng Han

**Affiliations:** 1 Changchun University of Traditional Chinese Medicine, 1035 Boshuo Road, Nanguan District, Changchun City, Jilin Province China; 2 Shandong University of Traditional Chinese Medicine, No.4655, Changqing University Science and Technology Park, Changqing District, Jinan City, Shandong Province China; 3 Affiliated Hospital of Changchun University of Traditional Chinese Medicine, No.1478 Gongnong Road, Chaoyang District, Changchun City, Jilin Province China

**Keywords:** Remnant preservation, Anterior cruciate ligament reconstruction, Systematic review, Meta-analysis, Overview

## Abstract

**Background:**

Anterior cruciate ligament injury is a common knee joint injury. Anterior cruciate ligament reconstruction is a common surgical treatment to treat anterior cruciate ligament injury. It may have certain advantages to retain the ligament stump during the operation, but the results of systematic evaluation on whether to retain the ligament stump are different. The conclusion is still controversial, and the quality needs to be strictly evaluated.

**Objective:**

To evaluate the methodological quality, risk of bias, reporting quality and evidence quality of the systematic review of remnant preservation in anterior cruciate ligament reconstruction, and to provide reference for clinical work.

**Methods:**

We systematically searched the system evaluations in 8 electronic databases, the languages were limited to Chinese and English, and the time limit was from the establishment of the database to June 2023. Two reviewers independently screened literature and extracted data. The methodological quality, risk of bias, reporting quality and quality of evidence were evaluated by AMSTAR-2, ROBIS, PRISMA and GRADE tools.

**Results:**

A total of 14 systematic reviews were included. The evaluation of results showed that the methodological quality of the included systematic reviews was relatively low, of which 5 were low quality and 9 were critically low quality. A small number of systematic reviews were low risk of bias. The system evaluation reports are relatively complete, but the lack of program registration is a common problem. A total of 111 pieces of clinical evidence were extracted from the included 14 systematic reviews. The quality of evidence was generally low, with only 7 pieces of high-quality evidence, 45 pieces of medium-quality evidence, and the rest were low and very low-quality evidence. Among the reasons for relegation, imprecision is the most common, followed by inconsistency and indirectness. The existing evidence shows that patients after anterior cruciate ligament reconstruction with remnant preservation have certain advantages in knee joint function, joint stability and proprioception recovery, which may be a more effective surgical method. However, it may also increase the incidence of postoperative complications and adverse reactions.

**Conclusion:**

Compared with Standard Technique, Remnant Preservation in Anterior Cruciate Ligament Reconstruction has more advantages in restoring joint function and stability and proprioception. But the potential risks should also be considered by surgeons. At present, the quality of evidence is generally low, and the reliability of the conclusion is insufficient. It still needs to be verified and further in-depth research is needed.

## Background

The anterior cruciate ligament (ACL) is an important ligament structure in the knee joint, which plays an important role in maintaining the stability of the knee joint, especially in the forward and anteromedial rotation of the knee joint [1–3]. ACL injury is a common knee joint injury, which is more common in young people [4, 5]. ACL injury may affect the normal flexion and extension of the knee joint, may affect the stability of the knee joint, may cause meniscus and articular cartilage injury, and even may have a negative impact on the proprioceptive function of the knee joint. [6, 7]. Studies have confirmed that ACL injury can greatly increase the risk of knee osteoarthritis [8, 9]. After complete rupture of the ACL, the ACL stump is gradually wrapped by synovial tissue. Although there is currently evidence to support that the injured ligament has a self-healing tendency under the action of cells and blood vessels, it is difficult to reconnect the two ends of the broken ligament because the ACL is surrounded by joint fluid, which causes great difficulties for cell invasion and remodeling. Therefore, the self-healing of ACL injury is poor. Anterior cruciate ligament reconstruction (ACLR) is a common surgical treatment [10]. Ligament reconstruction can restore the continuity of the ligament to the greatest extent, make the motor function of the knee joint recover faster, and reduce the risk of long-term secondary meniscus injury [4, 11]. In order to better expose the visual field and more accurately determine the location of the bone marrow canal, the surgeon generally chooses to thoroughly clean the ligament stump. However, in recent years, the preservation of ACL stump ACLR has received extensive attention. More and more studies have found that retaining ACL stump can promote the revascularization of grafts, obtain higher quadriceps muscle strength, and promote the recovery of joint stability and function [12–17]. Studies have also shown that preserving the ACL stump can promote tendon-bone healing and proprioceptive nerve remodeling after ACLR [18–21]. At present, there is still controversy about whether ACL stump should be retained and the relationship between ACL stump retention and tibial tunnel and cyclops lesion [22, 23]. Therefore, whether remnant preservation is superior to standard techniques in anterior cruciate ligament reconstruction requires higher-quality clinical evidence to guide clinical practice.

Systematic review and Meta-analysis (SR) are currently considered to be the highest grade of clinical evidence and play a guiding role in clinical practice. At present, there are several SRs to analyze the advantages of retaining ACL stump compared with standard surgery [24–26]. However, the methodological defects of SR may affect the final analysis results, and the risk of bias may also affect the credibility of the evidence. At the same time, the value of low-quality SR evidence may also be affected by exaggerating the effectiveness of interventions or selective reporting of adverse reactions [27]. Therefore, when we refer to the SR report, we should not only pay attention to the results, but also pay attention to its methodological quality, bias risk and report quality.

At present, there is still a lack of systematic evaluation overview of whether anterior cruciate ligament reconstruction with remnant preservation is superior to standard surgery. Therefore, we summarize the existing SRs on ACLR with remnant preservation, comprehensively summarize and review the methodological quality, bias risk, outcome indicators and evidence grade of existing SRs, in order to provide reference for clinicians, guideline makers and patients, and guide future high-quality SRs.

## Methods

### Protocol and registration

The protocol of this overview was registered on the International Prospective Register of Systematic Reviews (PROSPERO) (http://www.crd.york.ac.uk/prospero), registration number: CRD42023433774.

### Search strategy

We searched the Cochrane Library, Embase, Web of science, Medline (PubMed), China National Knowledge Infrastructure (CNKI), WanFang database, VIP database and Chinese Biology Medicine (CBM) database by computer. The search time range was from the establishment of the database to June 2023, and the search language was limited to Chinese and English. We also searched the research registry and grey literature, such as academic papers and conference reports. We used population (P), intervention (I), comparison (C) and study design (S) strategies for overview. Narrative reviews, SR summaries and comments are excluded. The specific retrieval strategies are as follows:

- P: Anterior Cruciate Ligament Injuries OR Anterior Cruciate Ligament Injury OR Anterior Cruciate Ligament Tear OR ACL Injury OR ACL Tear
- I: remnant preservation OR remnant OR stump OR ACL augmentation OR selective ACLR
- C: Anterior Cruciate Ligament Reconstruction OR ACLR OR ACL-R
- S: systematic review OR Meta-analysis

**Fig. 1:**
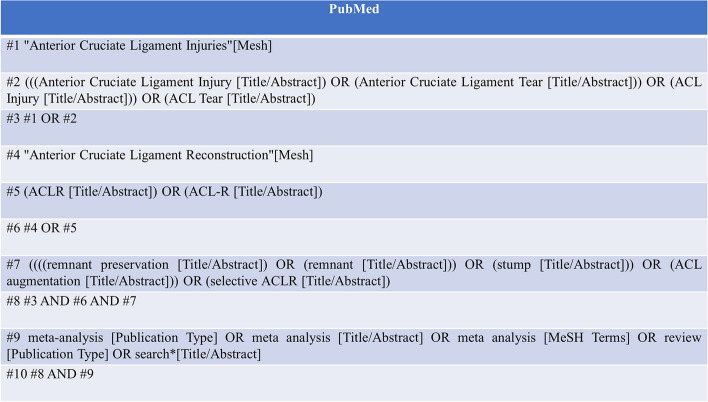
The search strategy of PubMed

The detailed search strategy of PubMed is shown in Fig. 1.

### Study selection and extraction

Two independent reviewers imported all retrieved studies into Endnote (X9), filtered duplicate studies, and filtered titles and abstracts. After cross-checking, the full text of the qualified study was further independently evaluated by two evaluators. If there were differences, the third reviewer participated.

Two reviewers independently extracted data, including: study characteristics (first author, year of publication, and country), subject characteristics (sample size), methodological characteristics (included study type, quality assessment tool, sensitivity analysis and subgroup analysis, publication bias) and results (number of studies included in SR, outcome indicators, adverse events, such as tibial tunnel enlargement and Cyclops lesions), and contacted study authors to obtain missing data. If there is a disagreement, the third examiner is involved.

### Assessment of methodological quality

A Measurement Tool to Assess Systematic Reviews 2 (AMSTAR 2) is an important assessment tool for systematic reviews, which can perform rapid and repeatable quality assessment of systematic reviews of randomized controlled trials, including defects that may be caused by improper review, and the items in AMSTAR 2 that deal with risk of bias identify domains specified in the Cochrane risk of bias instruments for randomized studies [28, 29]. Two reviewers independently assessed the methodological quality of SR inclusion. AMSTAR 2 was developed by AMSTAR and consists of 16 items, including 7 key projects (projects 2, 4, 7, 9, 11, 13, 15], which are used to conduct a critical assessment of the effectiveness of SR. According to the compliance with the standard, each project is evaluated as ‘yes’ (meeting the standard), ‘partly yes’ (partly meeting the standard) and ‘no’ (not meeting the standard) [28, 30].

### Assessment of risk of bias

The risk of bias for inclusion in SR was independently assessed by two reviewers using the Risk of Bias in Systematic reviews (ROBIS). The tool is divided into three stages to help determine the risk of bias in the review process, results and conclusions [31].

### Assessment of reporting quality

The quality of the reports included in the SR was independently assessed by two reviewers using the Preferred Reporting Items for Systematic Reviews and Meta-Analysis (PRISMA). PRISMA contains items critical to the transparent and complete reporting of SRs and is considered to be an evolution of the original Quality of Reporting of Meta-Analyses (QUOROM) guidelines. The PRISMA statement list covers 27 items in seven parts of SR: title, abstract, introduction, method, result, discussion and funding. Each item is described as ‘yes ', ‘partial yes’ and ‘no’, representing complete report, partial or incomplete report and missing report, respectively [32, 33].

### Assessment of evidence quality

The quality of evidence was independently assessed by two qualified reviewers using the Grades of Recommendations, Assessment, Development and Evaluation (GRADE) system (www.gradeworkinggroup.org), a widely used evidence quality rating tool. The quality of evidence was divided into ‘high’, ‘medium’, ‘low’ or ‘very low’. Evidence quality grading may be compromised due to five main aspects: the limitations of the study; inconsistency of the results, indirectness of the evidence, inaccuracy, and reporting bias [34].

### Data synthesis and presentation

A narrative synthesis was used in this overview. The characteristics and results of each SR as well as the results of AMSTAR 2, ROBIS and the PRISMA were summarized by tabulation and figures. The GRADE evidence profile and summary of findings table were generated by using the GRADE pro GDT online software (http://www.guidelinedevelopment.org/).

## Results

### Article selection results

**Fig. 2:**
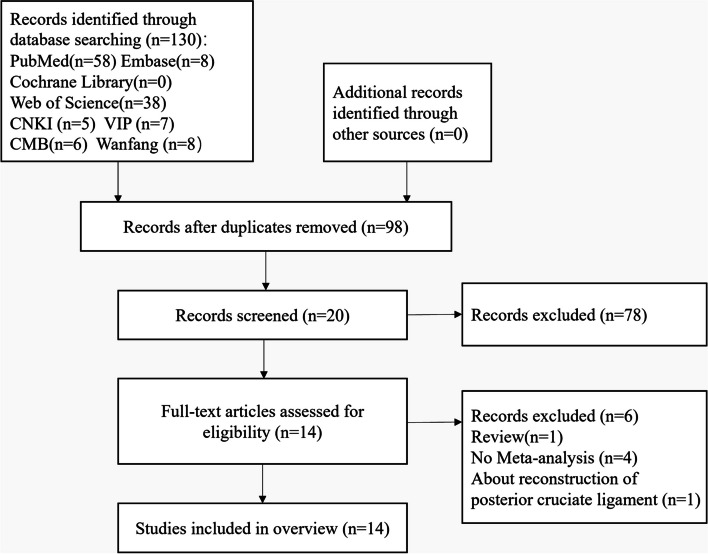
PRISMA flow chart for study selection

According to the above search strategy, 130 articles were initially retrieved, 32 duplicate articles were excluded by using the literature management software Endnote X9, 78 unrelated articles were excluded by reviewing the title and abstract, and 6 articles were excluded by reading the full text of the literature. Finally, 14 SRs were included [24–26, 35–45]. The specific article retrieval and exclusion process is shown in Fig. 2.

### Basic characteristics of selected articles

Of the 14 SRs, 7 were reported in English and 7 in Chinese. All SRs were published between 2016 and 2023. The authors are from China, Singapore and Italy. The 14 SRs included 5 to 15 original studies each. Four of the original research literatures included in SR were RCTs [26, 35–37]. All 14 SRs assessed the risk of bias in the original studies included in their analysis. One SR used PEDro [35], another used the JADAD scale [40], one used the modified Coleman methodology score (CMS) [24], one used the ROBINS-I [39], one used the Newcastle-Ottawa scale [38], and one used both the Newcastle-Ottawa scale (NOS) and the Cochrane bias risk assessment tool [25], Other SRs used the Cochrane Collaboration bias risk assessment tool [26, 36, 37, 42–45]. All 14 SRs included meta-analysis, and six of them performed sensitivity analysis [26, 35, 37, 40, 44, 45]. Furthermore, seven SRs included subgroup analysis [24, 25, 35, 37, 38, 40, 44]. Postoperative adverse reactions and complications were reported in 12 SRs [24–26, 35–38, 40–43, 45], 6 SRs assessed publication bias [26, 35, 37–39, 44], Sources of funding were reported in 7 SRs [25, 35, 40–43, 45]. The basic characteristics of all 14 SRs are shown in Table 1.

**Table 1:** Characteristics of included systematic reviews (SRs)

First author Year	Country	No. of include studies (sample size)	Type of included studies	Quality assessment tool	Data analysis methods	Sensitivity/subgroup analysis	Publication bias	Fund
Tie 2016 [35]	China	6(378)	RCT	PEDro	Meta-analysis	Y/Y	Y	Y
Zhang 2016 [43]	China	13(962)	RCT+NRCI	Cochrane	Meta-analysis	Y/N	N	Y
Chen 2016 [41]	China	5(295)	RCT	Cochrane	Meta-analysis	N/N	N	Y
Sun 2016	China	15(1192)	RCT+NRCI	Cochrane	Meta-analysis	Y/Y	Y	N
Ma 2017 [36]	China	6(346)	RCT	Cochrane	Meta-analysis	N/N	N	N
Zhang 2017	China	11(843)	RCT+NRCI	Cochrane	Meta-analysis	N/N	N	Y
Zhao 2017	China	15(1233)	RCT+NRCI	Jadad Scale	Meta-analysis	Y/Y	N	Y
Wang 2018 [37]	China	7(412)	RCT	Cochrane	Meta-analysis	Y/Y	Y	N
Wang 2019 [24]	China	11(1002)	RCT+NRCI	The modified Coleman methodology score(CMS)	Meta-analysis	N/Y	N	N
Huang 2021	China	8(531)	RCT	Cochrane	Meta-analysis	Y/N	Y	N
Xie 2022 [25]	China	10(777)	RCT+NRCI	Newcastle-Ottawa Scale、Cochrane	Meta-analysis	N/Y	N	Y
Yeo 2022 [38]	Singapore	11(1107)	RCT+NRCI	Newcastle-Ottawa Scale	Meta-analysis	N/Y	Y	N
Gu 2022	China	21(1584)	RCT+NRCI	Cochrane	Meta-analysis	Y/N	N	Y
Bosco 2023 [39]	Italy	7(472)	NRCI	ROBINS-I	Meta-analysis	N/N	Y	N

*RCT* Randomized controlled trials, *NRSI* Non-randomised studies of the effects of interventions

### Methodological quality of included SRs

Of the 14 SRs, 9 SRs were rated as critically Low quality [24, 25, 36, 39–43, 45], 5 SRs were rated as low quality [26, 35, 37, 38, 44]. The results of the methodological quality assessment using the AMSTAR 2 tool are shown in Table 2.

**Table 2:** Results of AMSTAR 2

Items	Tie 2016 [35]	Ma 2017 [36]	Wang 2018 [37]	Wang 2019 [24]	Huang 2021	Xie 2022 [25]	Yeo 2022 [38]	Bosco 2023 [39]	Gu 2022	Zhang 2016 [43]	Zhang 2017	Chen 2016 [41]	Sun 2016	Zhao 2017
1. Did the research questions and inclusion criteria for the review include the components of PICO?	Y	Y	Y	PY	Y	Y	Y	Y	PY	Y	Y	Y	Y	Y
2. Did the report of the review contain an explicit statement that the review methods were established prior to the conduct of the review and did the report justify any significant deviations from the protocol?	N	N	N	N	N	N	Y	Y	N	N	N	N	N	N
3. Did the review authors explain their selection of the study designs for inclusion in the review?	Y	Y	Y	N	Y	Y	Y	Y	Y	Y	Y	Y	Y	Y
4. Did the review authors use a comprehensive literature search strategy?	Y	PY	PY	PY	PY	PY	PY	PY	PY	PY	PY	PY	PY	PY
5. Did the review authors perform study selection in duplicate?	Y	N	Y	N	Y	Y	Y	Y	Y	N	Y	Y	Y	Y
6. Did the review authors perform data extraction in duplicate?	Y	Y	Y	N	Y	N	Y	N	N	N	Y	Y	Y	Y
7. Did the review authors provide a list of excluded studies and justify the exclusions?	Y	Y	Y	Y	Y	Y	Y	Y	Y	Y	PY	Y	Y	N
8. Did the review authors describe the included studies in adequate detail?	Y	Y	Y	Y	Y	Y	Y	Y	Y	Y	Y	Y	Y	Y
9. Did the review authors use a satisfactory technique for assessing the risk of bias in individual studies that were included in the review?	PY	Y	Y	Y	Y	Y	Y	Y	Y	Y	Y	Y	Y	Y
10. Did the review authors report on the sources of funding for the studies included in the review?	N	N	N	N	N	N	N	N	N	N	N	N	N	N
11. If meta-analysis was performed, did the review authors use appropriate methods for statistical combination of results?	Y	Y	Y	Y	Y	Y	Y	Y	Y	Y	Y	Y	Y	Y
12. If meta-analysis was performed, did the review authors assess the potential impact of risk of bias in individual studies on the results of the meta-analysis or other evidence synthesis?	Y	Y	Y	Y	Y	Y	Y	N	Y	Y	Y	Y	Y	Y
13. Did the review authors account for risk of bias in primary studies when interpreting/discussing the results of the review?	Y	Y	Y	Y	Y	Y	N	N	Y	Y	Y	Y	Y	Y
14. Did the review authors provide a satisfactory explanation for, and discussion of, any heterogeneity observed in the results of the review?	Y	N	Y	Y	Y	N	N	N	Y	Y	Y	Y	Y	Y
15. If they performed quantitative synthesis did the review authors carry out an adequate investigation of publication bias (small study bias) and discuss its likely impact on the results of the review?	Y	N	Y	N	Y	N	Y	N	N	N	N	N	Y	N
16. Did the review authors report any potential sources of conflict of interest, including any funding they received for conducting the review?	Y	Y	Y	Y	Y	Y	Y	Y	N	Y	Y	N	N	Y
**Methodological quality**	L	CL	L	CL	L	CL	L	CL	CL	CL	CL	CL	L	CL

*L* LOW, *CL* Critically Low

### Risk of bias of included SRs

According to ROBIS, all SRs (100 %) were at low risk in Phase I(assessment of relevance) and in Domain 1 (study eligibility criteria). For domain 2 (identification and selection of assessment studies), all SRs (100 %) were rated as high risk. 13 SRs (92.9 %) were rated as low risk in Domain 3 (data collection and research evaluation). [24–26, 35–37, 39–45]. 10 SRs ( 71.4 % ) were rated as low risk in Domain 4 ( synthesis and results ) [26, 35, 37, 38, 40–45], Eight cases ( 57.1 % ) were rated as low risk in Phase III ( risk of bias in review ) [26, 35, 37, 38, 40–42, 44]. The specific results are shown in Table 3.

**Table 3:** Results of Risk of Bias in Systematic reviews (ROBIS)

Review	Phase 1	Phase 2				Phase 3
	Assessing relevance	Domain 1. Study eligibility criteria	Domain 2. Identification and selection of studies	Domain 3. Data collection and study appraisal	Domain 4. Synthesis and findings	Risk of bias in the review
Tie 2016 [35]	L	L	H	L	L	L
Ma 2017 [36]	L	L	H	L	H	H
Wang 2018 [37]	L	L	H	L	L	L
Wang 2019 [24]	L	L	H	L	H	H
Huang 2021	L	L	H	L	L	L
Xie 2022 [25]	L	L	H	L	H	H
Yeo 2022 [38]	L	L	H	?	L	L
Bosco 2023 [39]	L	L	H	L	H	H
Gu 2022	L	L	H	L	L	H
Zhang 2016 [43]	L	L	H	H	L	H
Zhang 2017	L	L	H	L	L	L
Chen 2016 [41]	L	L	H	L	L	L
Sun 2016	L	L	H	L	L	L
Zhao 2017	L	L	H	L	L	L

*L* low risk, *H* high risk, *?* unclear risk

### Reporting quality of included SRs

**Fig. 3:**
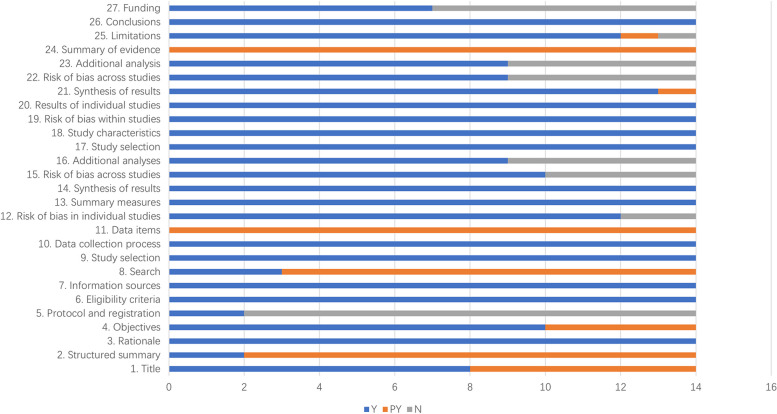
Results of PRISMA. ( Y: Yes PY: Partially Yes N: No)

Overall, the systematic reviews are comprehensive, but some reports have flaws. We found that some SRs did not report or did not fully report research programs and registrations (12/14, 85.7%), complete search strategy (11 / 14,78.6 %), sources of funding for included studies (14/14,100%), risk of publication bias (5/14, 35.7%), additional analysis (5/14, 35.7%) and funds (7/14, 50%). Only 2 SRs reported the design and registration of research programs in advance [38, 39]. At the same time, only two SRs reported a complete search strategy for a database, and reported other literature collection methods in addition to searching electronic databases [37, 41]. Most SRs did not report the risk of bias for research and additional analysis well, and only 7 SRs reported sources of funding and other funding [25, 35, 40–43, 45]. The PRISMA checklist for all SRs is shown in Table 4 and Fig. 3.

**Table 4:** Results of PRISMA

	Tie 2016 [35]	Ma 2017 [36]	Wang 2018 [37]	Wang 2019 [24]	Huang 2021	Xie 2022 [25]	Yeo 2022 [38]	Bosco 2023 [39]	Gu 2022	Zhang 2016 [43]	Zhang 2017	Chen 2016 [41]	Sun 2016	Zhao 2017
Title														
1. Title	Y	Y	Y	Y	Y	Y	Y	Y	PY	PY	PY	PY	PY	PY
Abstract														
2. Structured summary	PY	PY	PY	PY	PY	PY	PY	PY	PY	PY	PY	PY	Y	Y
Introduction														
3. Rationale	Y	Y	Y	Y	Y	Y	Y	Y	Y	Y	Y	Y	Y	Y
4. Objectives	Y	PY	Y	PY	Y	Y	PY	Y	PY	Y	Y	Y	Y	Y
Methods														
5. Protocol and registration	N	N	N	N	N	N	Y	Y	N	N	N	N	N	N
6. Eligibility criteria	Y	Y	Y	Y	Y	Y	Y	Y	Y	Y	Y	Y	Y	Y
7. Information sources	Y	Y	Y	Y	Y	Y	Y	Y	Y	Y	Y	Y	Y	Y
8. Search	Y	PY	Y	PY	PY	PY	PY	PY	PY	PY	PY	Y	PY	PY
9. Study selection	Y	Y	Y	Y	Y	Y	Y	Y	Y	Y	Y	Y	Y	Y
10. Data collection process	Y	Y	Y	Y	Y	Y	Y	Y	Y	Y	Y	Y	Y	Y
11. Data items	PY	PY	PY	PY	PY	PY	PY	PY	PY	PY	PY	PY	PY	PY
12. Risk of bias in individual studies	Y	Y	Y	N	Y	N	Y	Y	Y	Y	Y	Y	Y	Y
13. Summary measures	Y	Y	Y	Y	Y	Y	Y	Y	Y	Y	Y	Y	Y	Y
14. Synthesis of results	Y	Y	Y	Y	Y	Y	Y	Y	Y	Y	Y	Y	Y	Y
15. Risk of bias across studies	Y	N	Y	N	Y	N	Y	Y	N	Y	Y	Y	Y	Y
16. Additional analyses	Y	N	Y	Y	Y	Y	Y	N	Y	N	N	N	Y	Y
Results														
17. Study selection	Y	Y	Y	Y	Y	Y	Y	Y	Y	Y	Y	Y	Y	Y
18. Study characteristics	Y	Y	Y	Y	Y	Y	Y	Y	Y	Y	Y	Y	Y	Y
19. Risk of bias within studies	Y	Y	Y	Y	Y	Y	Y	Y	Y	Y	Y	Y	Y	Y
20. Results of individual studies	Y	Y	Y	Y	Y	Y	Y	Y	Y	Y	Y	Y	Y	Y
21. Synthesis of results	Y	Y	Y	Y	Y	Y	Y	Y	Y	Y	Y	PY	Y	Y
22. Risk of bias across studies	Y	N	Y	N	Y	N	Y	N	N	Y	Y	Y	Y	Y
23. Additional analysis	Y	N	Y	Y	Y	Y	Y	N	Y	N	N	N	Y	Y
Discussion														
24. Summary of evidence	PY	PY	PY	PY	PY	PY	PY	PY	PY	PY	PY	PY	PY	PY
25. Limitations	PY	Y	Y	Y	Y	Y	Y	Y	Y	Y	Y	Y	N	Y
26. Conclusions	Y	Y	Y	Y	Y	Y	Y	Y	Y	Y	Y	Y	Y	Y
Funding														
27. Funding	Y	N	N	N	N	Y	N	N	Y	Y	Y	Y	N	Y

*Y* Yes, *PY* Partially Yes, *N* No

### Evidence quality

A total of 20 outcome indicators were extracted from the included 14 SRs. We divided the outcome indicators into 6 categories: Clinical score, Joint function and stability, Postoperative proprioceptive sense, Graft status, Postoperative complications and adverse reactions, and operation time.

Clinical score: Postoperative Lysholm score, postoperative Tegner score, postoperative IKDC score, postoperative IKDC grade. Joint function and stability: postoperative KT1000 / 2000 joint measurement results, postoperative arthrometry side-to-side difference, postoperative Pivot shift test results, postoperative Lachman test results, postoperative knee flexion and extension activity (ROM). Postoperative proprioceptive sense: postoperative Proprioceptive sense difference, postoperative passive activity perception threshold (TTDPM), postoperative joint position sensation, postoperative passive angle regeneration test results, postoperative graft synovial coverage, postoperative tibial tunnel enlargement. Postoperative complications and adverse reactions: postoperative Cyclops lesions, postoperative complications, postoperative reinjury rate.

#### Clinical score

The evidence grade of all Clinical scores is shown in Table 5.

**Table 5:** Overview of clinical scoring evidence level

Clinical Score	Risk of bias	Inconsistency	Indirectness	Imprecision	Other considerations	Effect estimate (95% CI)	Certainty	References
Lysholm Score								
	0	0	-1	-1	0	MD 1.60(0.17,3.04)	Low	Tie 2016 [35]
	0	0	-1	-1	0	MD 2.07(0.85,3.28)	Low	Ma 2017 [36]
	0	0	-1	-1	0	MD 2.2(0.95,3.45)	Low	Wang 2018 [37]
	0	0	-1	0	0	MD 0.95(0.07,1.83)	Medium	Wang 2019 [24]
	0	-1	-1	0	0	MD 2.2(0.82,3.58)	Low	Huang 2021
	0	0	-1	0	0	MD 0.98(0.32,1.64)	Medium	Xie 2022 [25]
	0	-2	-1	-1	0	MD 1.25(-1.57,4.07)	Very Low	Yeo 2022 [38]
	0	-2	-1	-1	0	SMD 0.61(-0.49,1.71)	Very Low	Bosco 2023 [39]
	0	-1	-1	0	0	SMD 0.84(0.34,1.35)	Low	Gu 2022
	0	-2	-1	0	0	MD 2.45(0.52,4.39)	Very Low	Zhang 2016 [43]
	0	0	-1	0	0	MD 1.34(0.63,2.06)	Medium	Zhang 2017
	0	0	-1	-1	0	MD 1.47(-0.09,3.03)	Low	Chen 2016 [41]
	0	0	-1	-1	0	MD 0.20(-0.13, 0.53)	Low	Zhao 2017
< 1 month	0	0	-1	-1	0	SMD 0.18(-0.33,0.70)	Low	Sun 2016
3 months	0	-2	-1	0	0	SMD 1.62(0.42,2.81)	Very Low	Sun 2016
6 months	0	-2	-1	0	0	SMD 4.08(2.22,5.94)	Very Low	Sun 2016
9 months	0	-2	-1	0	0	SMD 4.68(1.67,7.69)	Very Low	Sun 2016
12 months	0	-2	-1	0	0	SMD 4.70(3.78,5.62)	Very Low	Sun 2016
>12 months	0	-2	-1	0	0	SMD 1.85(0.85,2.85)	Very Low	Sun 2016
Tenger Score								
	0	0	-1	-1	0	SMD -0.13(-0.47,0.22)	Low	Xie 2022 [25]
	0	0	-1	-1	0	MD 0.33(-0.41,1.08)	Low	Yeo 2022 [38]
	0	0	-1	-1	0	SMD 0.37(0.12,0.63)	Low	Bosco 2023 [39]
	0	0	-1	-1	0	SMD -0.02(-0.21,0.16)	Low	Gu 2022
IKDC Score								
	0	0	-1	-1	0	MD 0.07(-1.53,1.67)	Low	Tie 2016 [35]
	0	0	-1	-1	0	MD 0.24(-1.36,1.84)	Low	Ma 2017 [36]
	0	0	-1	-1	0	MD -0.34(-2.34,1.67)	Low	Wang 2018 [37]
	0	0	-1	-1	0	MD 0.07(-1.53,1.67)	Low	Wang 2019 [24]
	0	-1	-1	0	0	MD 2.28(1.2,3.37)	Low	Huang 2021
	0	0	-1	-1	0	MD 0.26(-1.1,1.61)	Low	Yeo 2022
	0	-1	-1	0	0	SMD 0.87(0.34,1.35)	Low	Gu 2022
	0	0	-1	-1	0	MD 1.28(0.27,2.28)	Low	Zhang 2017
	0	0	-1	-1	0	MD -0.18(-1.78,1.42)	Low	Chen 2016 [41]
3 months	0	-1	-1	0	0	SMD 2.12(1.30,2.93)	Low	Sun 2016
6 months	0	-2	-1	0	0	SMD 4.22(2.13,6.31)	Very Low	Sun 2016
9 months	0	-2	-1	0	0	SMD 5.72(3.19,8.24)	Very Low	Sun 2016
12 months	0	-2	-1	0	0	SMD 2.51(0.79,4.24)	Very Low	Sun 2016
>12 months	0	-2	-1	0	0	SMD 1.18(0.02,2.35)	Very Low	Sun 2016
IKDC Grade								
	0	0	-1	-1	0	RR 1.158(0.978,1.372)	Low	Tie 2016 [35]
	0	0	-1	-1	0	OR 2.09(0.73,5.97)	Low	Ma 2017 [36]
	0	0	-1	-1	0	RR 1.05(0.96,1.14)	Low	Wang 2018 [37]
	0	0	-1	-1	0	OR 2.05(0.70,5.97)	Low	Wang 2019 [24]
	0	0	-1	0	0	OR 2.19(1.03,4.65)	Medium	Xie 2022 [25]
	0	0	-1	-1	0	OR 1.18(0.57,2.43)	Low	Yeo 2022 [38]

A total of 14 SRs were included in the meta-analysis of postoperative Lysholm score. A total of 19 pieces of clinical evidence were formed, including 3 pieces in the high grade of evidence, 8 pieces in the low grade of evidence, and 8 pieces in the very low grade of evidence. The reasons for the degradation of the evidence grade are mainly due to the indirectness (100 %) of the outcome evaluation of the Lysholm score as the outcome index, the large heterogeneity (43.8 %) when merging the data, and the inaccuracy (50 %) caused by the failure to reach the minimum sample size or the excessive confidence interval.

Meta-analysis of postoperative Tegner score was performed in 4 SRs [25, 38, 39, 45]. A total of 4 pieces of clinical evidence were formed, all of which were low-grade evidence. The reasons for the degradation of the evidence grade are mainly due to the indirectness of the Tegner score (100 %) and the inaccuracy caused by the failure to reach the minimum sample size or the excessive confidence interval (100 %).

There were 10 SRs for meta-analysis of postoperative IKDC scores [24, 26, 35–38, 41, 42, 44, 45], and a total of 14 pieces of clinical evidence were formed, including 10 pieces of low-grade evidence (71.4 %) and 4 pieces of very low-grade evidence (28.6 %). The reasons for the degradation of the evidence grade are mainly due to the indirectness (100 %) of the IKDC score, the large heterogeneity (50 %) when merging the data, and the inaccuracy (50 %) caused by the failure to reach the minimum sample size or the excessive confidence interval.

6 SRs performed a meta-analysis of postoperative IKDC grade [24, 25, 35–38]. A total of 6 pieces of clinical evidence were formed, including 5 pieces of low-grade evidence (83.3 %) and 1 piece of very low-grade evidence (16.7 %). The reasons for the downgrading of the evidence grade are mainly due to the indirectness of the IKDC grade (100 %), the large heterogeneity (50 %) when merging the data, and the inaccuracy (83.3 %) caused by the failure to reach the minimum sample size or the excessive confidence interval.

#### Joint function and stability

The evidence grade of postoperative knee joint stability and function are summarized in Table 6.

**Table 6:** Overview of evidence levels related to joint function and stability

Outcome	Risk of bias	Inconsistency	Indirectness	Imprecision	Other considerations	Effect estimate (95% CI)	Certainty	References
KT1000 / 2000 measurements								
	0	-1	0	-1	0	MD 0.24(-0.69,0.2)	Low	Tie 2016 [35]
	0	-1	0	-1	0	SMD -0.52(-0.94,-0.11)	Low	Ma 2017 [36]
	0	-1	0	0	0	MD -0.36(-0.57,-0.15)	Medium	Wang 2019 [24]
	0	0	0	-1	0	MD -0.29(-0.52,-0.06)	Medium	Huang 2021
	0	0	0	0	0	SMD -0.22(-0.42, -0.03)	High	Xie 2022 [25]
	0	-1	0	-1	0	SMD 0.17(-0.22,0.57)	Low	Bosco 2023 [39]
	0	0	0	0	0	SMD -0.2(-0.39, -0.01)	High	Gu 2022
	0	-1	0	-1	0	SMD -0.28(-0.76,0.2)	Low	Zhang 2016 [43]
	0	0	0	-1	0	MD -0.05(-0.13, 0.03)	Medium	Zhang 2017
	0	0	0	-1	0	MD -0.36(-0.63, -0.1)	Medium	Chen 2016 [41]
	0	0	0	0	0	MD -0.21(-0.25,-0.16)	High	Zhao 2017
3 months	0	0	0	-1	0	SMD -0.34(-0.63, -0.05)	Medium	Sun 2016
6 months	0	-1	0	-1	0	SMD -0.85(-1.29--0.42)	Low	Sun 2016
9 months	0	0	0	-1	0	SMD -1.54(-1.87, -1.21)	Medium	Sun 2016
12 months	0	-1	0	-1	0	SMD -0.87(-1.71, -0.04)	Low	Sun 2016
>12 months	0	-1	0	-1	0	SMD -1.51(-2.15, -0.87)	Low	Sun 2016
Arthrometry side-to-side difference								
	0	-2	0	-1	0	MD -0.71(-0.87,-0.55)	Very Low	Wang 2018 [24]
	0	0	0	0	0	MD -0.33(-0.47,-0.18)	High	Yeo 2022 [38]
Pivot shift test								
	0	-1	0	-1	0	RR 1.00(0.87,1.15)	Low	Tie 2016 [35]
	0	0	0	-1	0	OR 0.52(0.24,1.13)	Medium	Ma 2017 [36]
	0	0	0	-1	0	RR 1.06(0.97,1.17)	Medium	Wang 2018 [37]
	0	0	0	-1	0	OR 0.96(0.44,2.10)	Medium	Wang 2019 [24]
	0	0	0	-1	0	OR 1.06(0.46,2.45)	Medium	Huang 2021
	0	0	0	-1	0	OR 1.52(0.99,2.34)	Medium	Yeo 2022 [38]
	0	0	0	-1	0	RR 1.07(0.94,1.21)	Medium	Chen 2016 [41]
	0	0	0	0	0	RR 1.10(1.01,1.19)	High	Sun 2016
Lachman test								
	0	0	0	-1	0	RR 1.04(0.87,1.23)	Medium	Tie 2016 [35]
	0	0	0	-1	0	OR 0.76(0.35,1.68)	Medium	Ma 2017 [36]
	0	0	0	-1	0	RR 1.04(0.87,1.23)	Medium	Wang 2018 [37]
	0	0	0	-1	0	OR 0.59(0.28,1.23)	Medium	Huang 2021
	0	0	0	-1	0	OR 1.66(0.79,3.49)	Medium	Xie 2022 [25]
	0	0	0	-1	0	OR 1.71(0.98,3.00)	Medium	Yeo 2022 [38]
	0	0	0	-1	0	RR -0.03(-0.10,0.05)	Medium	Gu 2022
	0	0	0	-1	0	RR 1.04(0.87,1.23)	Medium	Chen 2016 [41]
	0	0	0	-1	0	RR 1.07(0.99.1.16)	Medium	Sun 2016
ROM								
	0	0	0	-1	0	MD -0.53(-2.49,1.43)	Medium	Ma 2017 [36]
	0	0	0	-1	0	SMD 0.27(-0.13,0.68)	Medium	Xie 2022 [25]
	0	0	0	-1	0	MD -0.55(-2.47,1.37	Medium	Chen 2016 [41]

A total of 12 SRs conducted a meta-analysis of the postoperative KT1000 / 2000 arthrometer measurement results [24–26, 35, 36, 39–45], and a total of 16 pieces of clinical evidence were formed, including 3 pieces of high-grade evidence (18.9 %), 6 medium-grade data (37.5 %), and 7 pieces of low-grade evidence (43.8 %). The main reasons for the downgrading of the evidence grade are the large heterogeneity (50 %) when merging the data and the inaccuracy (75 %) caused by the failure to reach the minimum sample size or the excessive confidence interval.

Meta-analysis of postoperative arthrometry side-to-side difference was performed in 2 SRs [37, 38], resulting in 2 pieces of clinical evidence, including 1 piece of high-grade evidence (50 %) and 1 piece of very low-grade evidence (50 %). The main reasons for the degradation of very low-grade evidence are the large heterogeneity when merging data and the inaccuracy caused by not reaching the minimum sample size or too large confidence interval.

8 SRs performed a meta-analysis of the postoperative Pivot shift test results [24, 26, 35–38, 41, 44], and a total of 8 pieces of clinical evidence were formed, including 1 piece of high-grade evidence (12.5 %), 6 pieces of medium-grade evidence (75 %), and 1 piece of low-grade evidence (12.5 %). The main reasons for the degradation of the evidence grade are the large heterogeneity (12.5 %) when merging the data and the inaccuracy (87.5 %) caused by the failure to reach the minimum sample size or the excessive confidence interval.

Meta-analysis of postoperative Lachman test results was performed in 9 SRs [25, 26, 35–38, 41, 44, 45]. A total of 9 pieces of clinical evidence were formed, all of which were medium-grade evidence. The reason for the degradation of the evidence grade is the inaccuracy (100 %) caused by the failure to reach the minimum sample size or the excessive confidence interval.

Meta-analysis of postoperative knee flexion and extension activity (ROM) was performed in 3 SRs [25, 36, 41]. A total of 3 pieces of clinical evidence were formed, all of which were medium-grade evidence. The reason for the degradation of the evidence grade is the inaccuracy (100 %) caused by the failure to reach the minimum sample size or the excessive confidence interval.

#### Postoperative proprioceptive sense

An overview of the evidence grade for postoperative proprioceptive sense is shown in Table 7.

**Table 7:** Overview of the level of evidence related to postoperative proprioceptive sense

Outcome	Risk of bias	Inconsistency	Indirectness	Imprecision	Other considerations	Effect estimate (95% CI)	Certainty	References
Proprioception difference								
	0	-1	0	0	0	MD 0.41(0.13,0.69)	Medium	Huang 2021
	0	-2	0	-1	0	SMD -1.72(-3.32, -0.13)	Very Low	Zhang 2016 [43]
3 months	0	-1	0	-1	0	MD -0.72(-1.16, -0.28)	Low	Zhao 2017
12 months	0	-1	0	0	0	MD -0.45(-0.66, -0.25)	Medium	Zhao 2017
TTDPM								
	0	-1	0	0	0	SMD -1.15(-1.77, -0.52)	Medium	Gu 2022
	0	-1	0	0	0	MD -0.5(-0.74, -0.26)	Medium	Zhang 2017
Joint position feeling								
	0	0	0	-1	0	MD -0.3(-0.79,0.18)	Medium	Zhang 2017
Passive angle regeneration test								
	0	0	0	0	0	MD -0.13(-0.26, -0.01)	High	Zhang 2017

3 SRs conducted a meta-analysis of postoperative Proprioceptive sense [26, 40, 43]. A total of 4 pieces of clinical evidence were formed, including 2 pieces of medium-grade evidence (50 %), 1 piece of low-grade evidence (25 %), and 1 piece of very low-grade evidence (25 %). The main reasons for the degradation of the evidence grade are the large heterogeneity (100 %) and the inaccuracy (50 %) caused by the failure to reach the minimum sample size or the excessive confidence interval.

2 SRs conducted a meta-analysis of postoperative passive motion perception threshold (TTDPM) to form two pieces of clinical evidence [42, 45], both of which were medium-grade evidence. The reason for the downgrading of the evidence grade is that there is a large heterogeneity (100 %) when merging data.

1 SR performed a meta-analysis of postoperative joint position sensation [42], and a total of 1 piece of clinical evidence was formed, which was medium-grade evidence. The reason for the degradation of the grade of evidence is the imprecision (100 %) caused by not reaching the minimum sample size or too large confidence interval.

1 SR conducted a meta-analysis of the results of postoperative passive angle regeneration test [42], and a total of 1 piece of clinical evidence was formed, which was high-grade evidence.

#### Graft status

The evidence grade of postoperative Graft status is shown in Table 8.

**Table 8:** Overview of evidence level related to graft status

Outcome	Risk of bias	Inconsistency	Indirectness	Imprecision	Other considerations	Effect estimate (95% CI)	Certainty	References
Coverage of synovium								
	0	0	0	-1	0	OR 1.55(0.66-3.65)	Medium	Xie 2022 [25]
	0	0	0	-1	0	RR 0.27(0.16,0.39)	Medium	Gu 2022
	0	-2	0	-1	0	RR 0.3(-0.3,0.9)	Very Low	Zhang 2016 [43]
Expansion of tibial tunnel								
	0	-1	0	-1	0	MD 5.56(1.81,9.49)	Low	Tie 2016 [35]
	0	0	0	-1	0	SMD -0.85(-1.29,-0.42)	Medium	Ma 2017 [36]
	0	-1	0	-1	0	MD -2.51(-5.84,0.82)	Low	Huang 2021
	0	0	0	-1	0	SMD -0.66(-1.08, -0.23)	Medium	Xie 2022 [25]
	0	0	0	-1	0	RR -0.15(-0.26, -0.04)	Medium	Gu 2022
	0	0	0	-1	0	SMD -0.66(-1.08, -0.23)	Medium	Zhang 2016 [43]
	0	-1	0	-1	0	MD -0.44(-0.71, -0.16)	Low	Chen 2016 [41]
Status of Graft								
	0	-1	0	-1	0	OR 2.67(1.06,6.71)	Low	Wang 2019 [24]

3 SRs performed a meta-analysis of postoperative graft synovial coverage [25, 43, 45]. A total of 3 pieces of clinical evidence were formed, including 2 pieces of medium-grade evidence (66.7 %) and 1 piece of very low-grade evidence (33.3 %). The reasons for the degradation of the evidence grade are mainly due to the large heterogeneity (25 %) when merging the data, and the inaccuracy (100 %) caused by the failure to reach the minimum sample size or the excessive confidence interval.

Meta-analysis of postoperative tibial tunnel enlargement was performed in 7 SRs [25, 26, 35, 36, 43, 45]. A total of 7 pieces of clinical evidence were formed, including 4 pieces of medium-grade evidence (57.1 %) and 3 pieces of low-grade evidence (42.9 %). The main reasons for the degradation of the evidence grade are the inaccuracy (100 %) caused by the failure to reach the minimum sample size or the excessive confidence interval and the large heterogeneity (42.9 %) when merging the data.

1 SR performed a meta-analysis of graft status [24]. A total of 1 piece of clinical evidence was formed, which was low-grade evidence. The reason for the degradation of the evidence grade is the inaccuracy caused by the failure to reach the minimum sample size or the excessive confidence interval and the large heterogeneity when merging the data.

#### Postoperative complications and adverse reactions

The evidence grade of postoperative complications and adverse reactions are shown in Table 9.

**Table 9:** The level of evidence related to postoperative complications and adverse reactions

Outcome	Risk of bias	Inconsistency	Indirectness	Imprecision	Other considerations	Effect estimate (95% CI)	Certainty	References
Cyclops lesion								
	0	0	0	-1	0	RR 1.51 (0.84,2.70)	Medium	Tie 2016 [35]
	0	0	0	-1	0	OR 1.84(0.87,4.35)	Medium	Ma 2017 [36]
	0	-1	0	-1	0	OR 3.92(0.53,29.29)	Low	Xie 2022 [25]
	0	0	0	-1	0	OR 1.35(0.63,2.90)	Medium	Zhang 2016 [43]
	0	0	0	-1	0	RR 1.12(0.36,3.52)	Medium	Zhao 2017
Rate of complications								
	0	-1	0	-1	0	RR 0.95(0.62,1.46)	Low	Wang 2018 [37]
	0	0	0	-1	0	OR 1.24(0.76,2.02)	Medium	Wang 2019 [24]
	0	0	0	-1	0	OR 1.33(0.46,3.90)	Medium	Yeo 2022 [38]
Re-injury rate								
	0	0	0	-1	0	OR 0.57(0.18,1.74)	Medium	Xie 2022 [25]

Meta-analysis of the incidence of postoperative Cyclops lesions was performed in 5 SRs [25, 35, 36, 40, 43], A total of 5 pieces of clinical evidence were formed, including 4 pieces of medium-grade evidence (80 %) and 1 piece of low-grade evidence (20 %). The main reasons for the degradation of the evidence grade are the inaccuracy (100 %) caused by the failure to reach the minimum sample size or the excessive confidence interval and the large heterogeneity (20 %) when merging the data.

3 SRs performed a meta-analysis of postoperative complications [24, 37, 38], A total of 3 pieces of clinical evidence were formed, including 2 pieces of medium-grade evidence (66.7 %) and 1 piece of low-grade evidence (33.3 %). The main reason for the degradation of the evidence grade is the inaccuracy (100 %) caused by the failure to reach the minimum sample size or the excessive confidence interval and the large heterogeneity (33.3 %) when merging the data.

1 SR conducted a meta-analysis of postoperative re-injury rate [25]. A total of 1 piece of clinical evidence was formed, which was medium-grade evidence. The main reason for the degradation of the evidence grade is the inaccuracy (100 %) caused by the failure to reach the minimum sample size or the excessive confidence interval.

#### Operation time

The evidence grade of postoperative complications and adverse reactions is shown in Table 10.

**Table 10:** Overview of evidence level of operation time

Outcome	Risk of bias	Inconsistency	Indirectness	Imprecision	Other considerations	Effect estimate (95% CI)	Certainty	References
Operation time								
	0	0	0	0	0	MD 11.69(8.85,14,54)	High	Xie 2022 [25]
	0	-2	0	-1	0	MD -4.49(-38.85,29.88)	Very Low	Zhao 2017

2 SRs conducted a meta-analysis of postoperative re-injury rate [25, 40]. A total of 2 pieces of clinical evidence was formed, including 1 piece of high-grade evidence, and 1 piece of very low-grade data (50 %). The reason for the degradation of very low-grade evidence is the inaccuracy caused by the failure to reach the minimum sample size or the excessive confidence interval and the large heterogeneity when merging data.

## Discussion

With the development of evidence-based medicine, many SRs have made a positive evaluation of the clinical effect of anterior cruciate ligament reconstruction with remnant preservation. However, the quality of SR seriously affects the reliability of its evidence, so the review of existing SR is of great significance for us to draw on its clinical evidence. At present, AMSTAR-2, ROBIS, PRISMA and GRADE are widely used in the review of SR to comprehensively review the methodological quality, bias risk, reporting quality and evidence quality of SR, respectively. The rigor of the review are widely recognized. By systematically summarizing and reviewing the existing SRs, we can effectively summarize the existing clinical evidence and provide targeted guidance for higher-quality SRs in the future. At the same time, the existing clinical evidence is summarized to improve the credibility of clinical evidence, and to better provide reference for the clinical practice of surgeons, so as to formulate a more scientific, rigorous and effective surgical treatment plan.

Our study systematically reviewed the existing 14 SRs on the clinical effect of anterior cruciate ligament reconstruction with remnant preservation. We found that according to the clinical evidence provided by the existing SR, there are certain advantages in retaining the stump in the anterior cruciate ligament reconstruction. Retaining the stump can effectively enhance the stability of the knee joint and promote the recovery of the proprioception of the knee joint. But it also has the disadvantages of high incidence of postoperative complications and adverse events and long operation time. However, the quality of the existing SR is generally low, the grade of evidence is not high, and there are still many problems. It is challenging to find clinical evidence that is truly convincing. Therefore, we cannot make an accurate judgment on the clinical effect of anterior cruciate ligament reconstruction with remnant preservation.

AMSTAR-2 is an internationally recognized and most widely used SR methodological quality evaluation tool. Its developers recommend focusing on whether there are methodological defects in key items and evaluating the overall quality of SR accordingly [28]. After evaluating the methodological quality of the included SRs through the AMSTAR-2 scale, we found that the included SRs had the following common problems: (1) Most of the SRs did not formulate pre-programs and register programs; (2) Most of the SR literature retrieval methods are single, and there is no supplementary retrieval and gray literature retrieval; (3) All SRs described the sources of funding for the included studies. (4) Most SRs did not test for publication bias. The final methodological quality evaluation results showed that all SRs were low or critically low quality studies.

ROBIS is currently the most advanced SR bias risk assessment tool. It can not only assess the risk of bias in the process of SR production and result interpretation, but also evaluate the correlation between SR and the practical problems to be solved by its users [31]. After evaluating the bias risk of the included SRs, we found that all SRs clearly studied the problem according to the PICOS principle and matched the problem to be solved with the target problem. At the same time, most SRs try to avoid the risk of bias in the process of research data extraction and quality evaluation. The source of bias risk was mainly that no other methods other than electronic database retrieval were used to determine the relevant research in the process of research retrieval and screening, which was consistent with the review results of AMSTAR-2 tool. In addition, the lack of sensitivity analysis is also a major problem in the risk of bias in some SRs during data synthesis.

The PRISMA checklist provides a reference for standardized writing and reporting of systematic reviews and meta-analyses [27, 33]. Standardized reporting can reduce the bias between actual research results and published results and increase the transparency of articles. Comparing the reporting specifications of the PRISMA checklist, we found that the lack of program registration, insufficient retrieval methods, insufficient extraction of data items, and lack of reporting of funding sources are the main reasons for insufficient reporting.

GRADE evidence classification is a common and popular method for evaluating the quality of evidence, which divides the quality of evidence group into four categories: high, medium, low and very low [34]. After evaluating the evidence by using the GRADE evidence grading system, we found that the grade of evidence for outcome indicators ranged from high to very low. The main reasons for the generally low grade of evidence are the indirectness of the outcome indicators, the large heterogeneity when merging the data, and the inaccuracy caused by the failure to reach the minimum sample size or the excessive confidence interval. The fundamental reason is that the original studies included in SR have defects in sample size calculation, random allocation method, allocation concealment, blind method and core outcome index setting.

Based on the above review results, we recommend that in the future, we should strictly design large-sample, multi-center randomized controlled trials in strict accordance with the requirements of the CONSORT statement. We suggest that we should improve the scientific nature of the research design, strengthen the understanding of the sample size, carry out large sample and multi-center clinical research, pay attention to the use of blind method and the use of objective outcome indicators in the research process, and ensure the representativeness of the participants. In addition, strict inclusion and exclusion criteria, stratified design analysis, control of the combined effects of confounding factors, and reduction of bias in clinical studies are also crucial.

At the same time, we should also recommend that researchers should follow the requirements of AMSTAR-2, ROBIS, PRISMA and GRADE when performing SR, especially to register or publish research programs on PROSPERO (http://www.crd.york.ac.uk/prospero) or Cochrane in advance to achieve transparency in the SR production process. In addition, attention should be paid to mentioning conflicts of interest or financial issues in the report. It is also necessary to conduct subgroup analysis or meta-regression analysis in the case of significant heterogeneity.

The existing evidence shows that patients after anterior cruciate ligament reconstruction with remnant preservation have certain advantages in knee joint function, joint stability and proprioception recovery, which may be a more effective surgical method. However, it may also increase the incidence of postoperative complications and adverse reactions. These potential risks should also be considered by surgeons.

## Limitations

There are some limitations in this study. (1) This study only includes Chinese and English literature, which may miss some research in other languages, and there may be language bias. (2) The evaluation process of this study is subjectively evaluated by researchers according to the evaluation criteria, so the evaluation results may be affected by the grade of researchers and the differences in understanding; (3) At present, there is no clear specification and standard for the evaluation method of SR, so our evaluation may not be comprehensive and accurate.

## Conclusions

Compared with standard technique, remnant preservation in anterior cruciate ligament reconstruction has more advantages in restoring joint function and stability and proprioception. However, it may also increase the incidence of postoperative complications and adverse reactions. These potential risks should also be considered by surgeons. At present, the quality of evidence is generally low, and the reliability of the conclusion is insufficient. It still needs to be verified and further research is needed.

## Data Availability

All data generated or analyzed during this study are included in this published article.

## Abbreviations

ACL: Anterior cruciate ligament
ACLR: Anterior cruciate ligament reconstruction
SR: Systematic review
